# Supplementary material for: L-type calcium channels regulate filopodia stability and cancer cell invasion downstream of integrin signalling
Source: Nat Commun. 2016 Dec 2;7:13297. doi: 10.1038/ncomms13297 (PMC5146291; doi:10.1038/ncomms13297)
Supplement: Supplementary Information — Supplementary Figures 1-12 and Supplementary Tables 1-2 [file ncomms13297-s1.pdf]

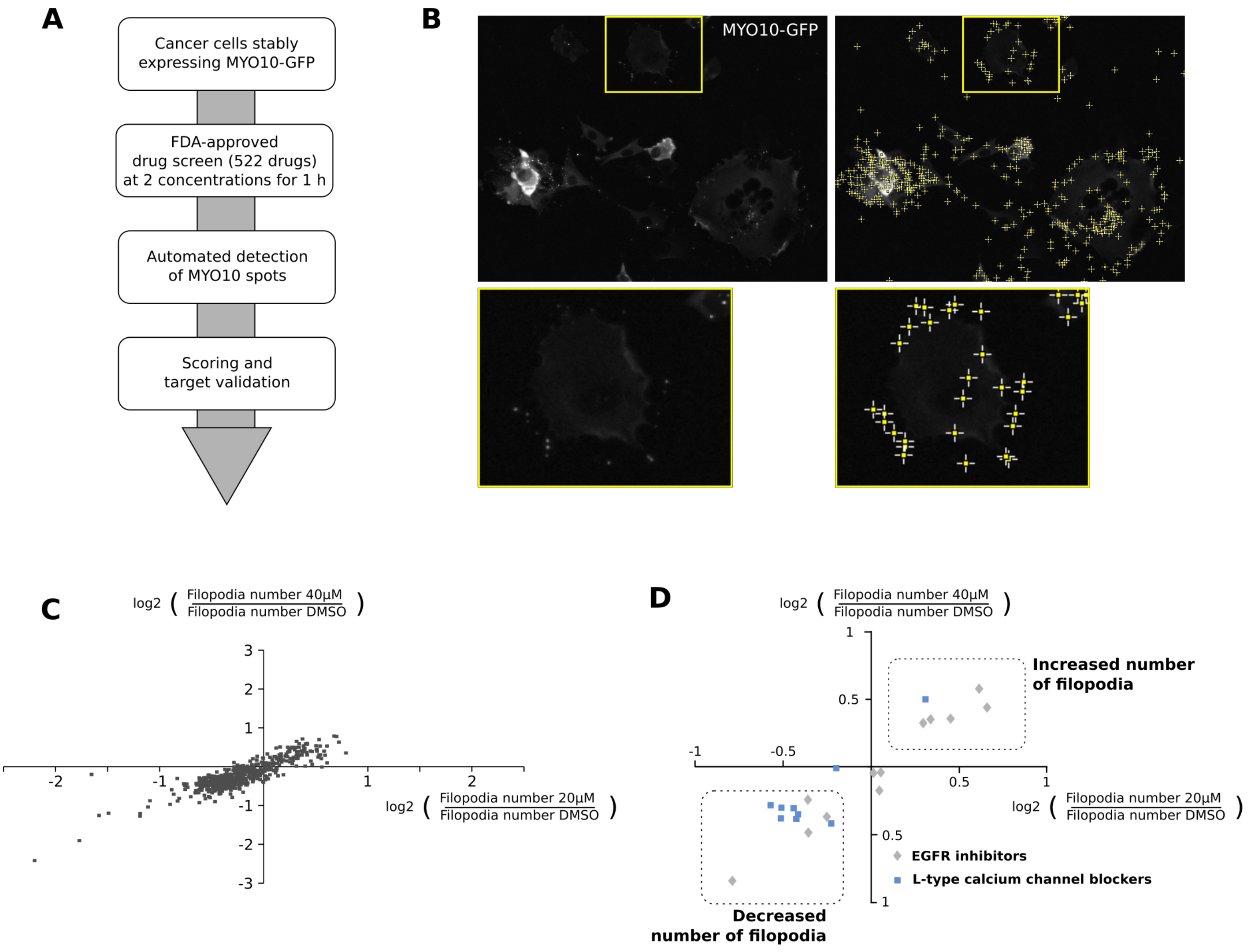

Supplementary Figure 1: An FDA-approved drug screen to identify regulators of filopodia formation
A: Work-flow of the FDA-approved drug screen used to identify novel regulators of filopodia formation. **B:** Representative images showing how MYO10-positive filopodia were automatically detected using an ImageJ-based macro (see method for details). The macro is provided as a supplementary file. **C:** The results of the drug screen displayed as a scatter plot. Each dot represents an individual inhibitor. **D:** The results of the drug screen highlighting the distribution of the various calcium channel blockers and EGFR inhibitors.

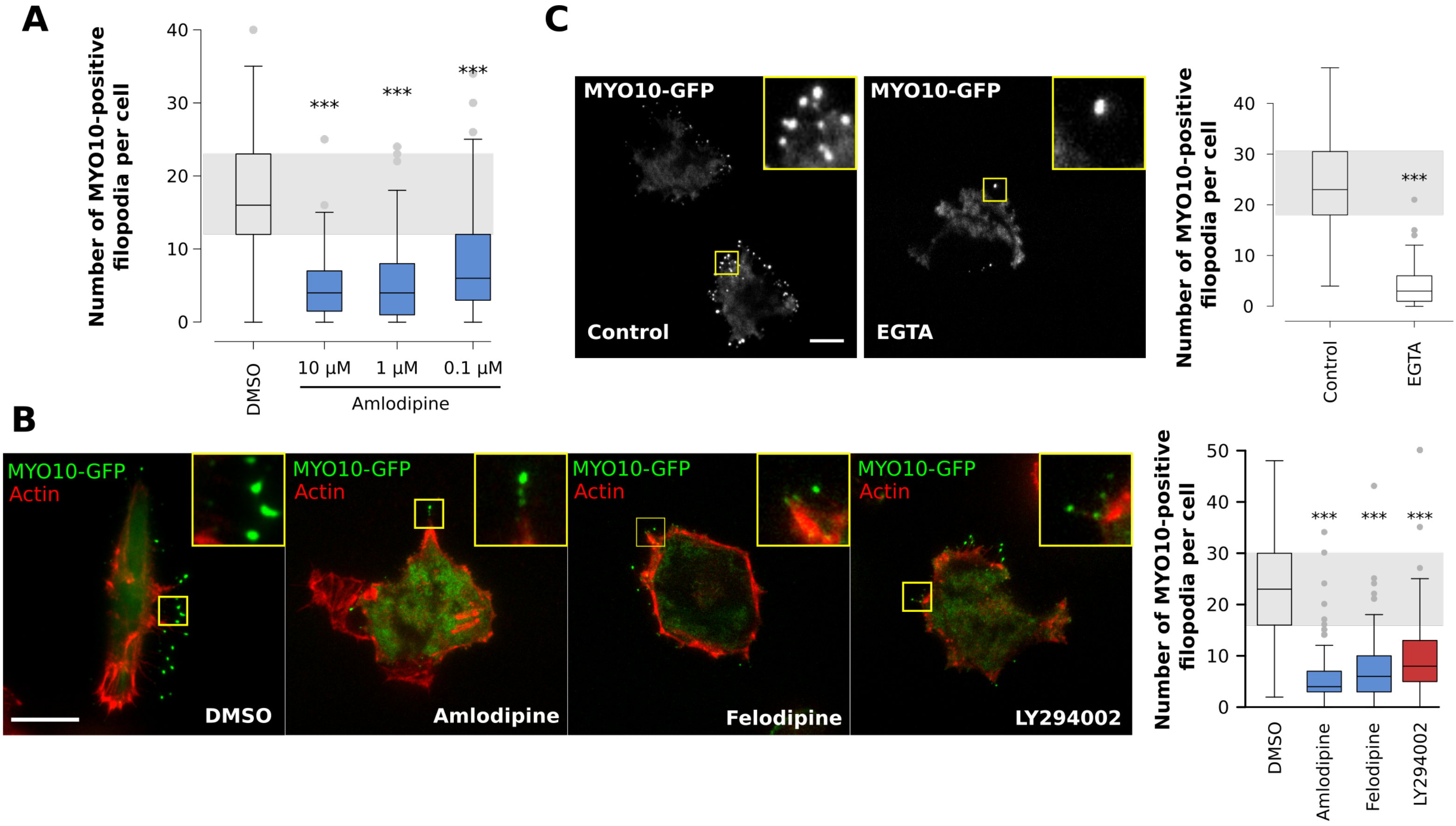

Supplementary Figure 2: Calcium entry via L-type calcium channels regulates filopodia formation

A: MDA-MB-231 cells transiently expressing MYO10-GFP and adhering to FN were treated with decreasing concentrations of Amlodipine (1 h), fixed, stained for actin and imaged on a TIRF microscope. The number of MYO10-positive filopodia was counted for each cell and displayed as a box plot (n > 150 cells, three biological repeats; *** p value < 4.22x10^{-22}). **B:** P53R172H PDAC cells transiently expressing MYO10-GFP were plated on FN, treated with DMSO, felodipine, amlodipine besylate or LY294002 (10 µM), fixed and the number of filopodia per cell was quantified (n > 94 cells, three biological repeats; scale bar = 20 µm; *** p value < 4.08x10^{-21}). **C:** MDA-MB-231 cells transiently expressing MYO10-GFP were plated on FN, treated with EGTA (2 mM) for 1 h, fixed and the number of filopodia per cell was quantified (n > 88 cells, three biological repeats; scale bar = 20 µm; *** p value < 2.3x10^{-36}).

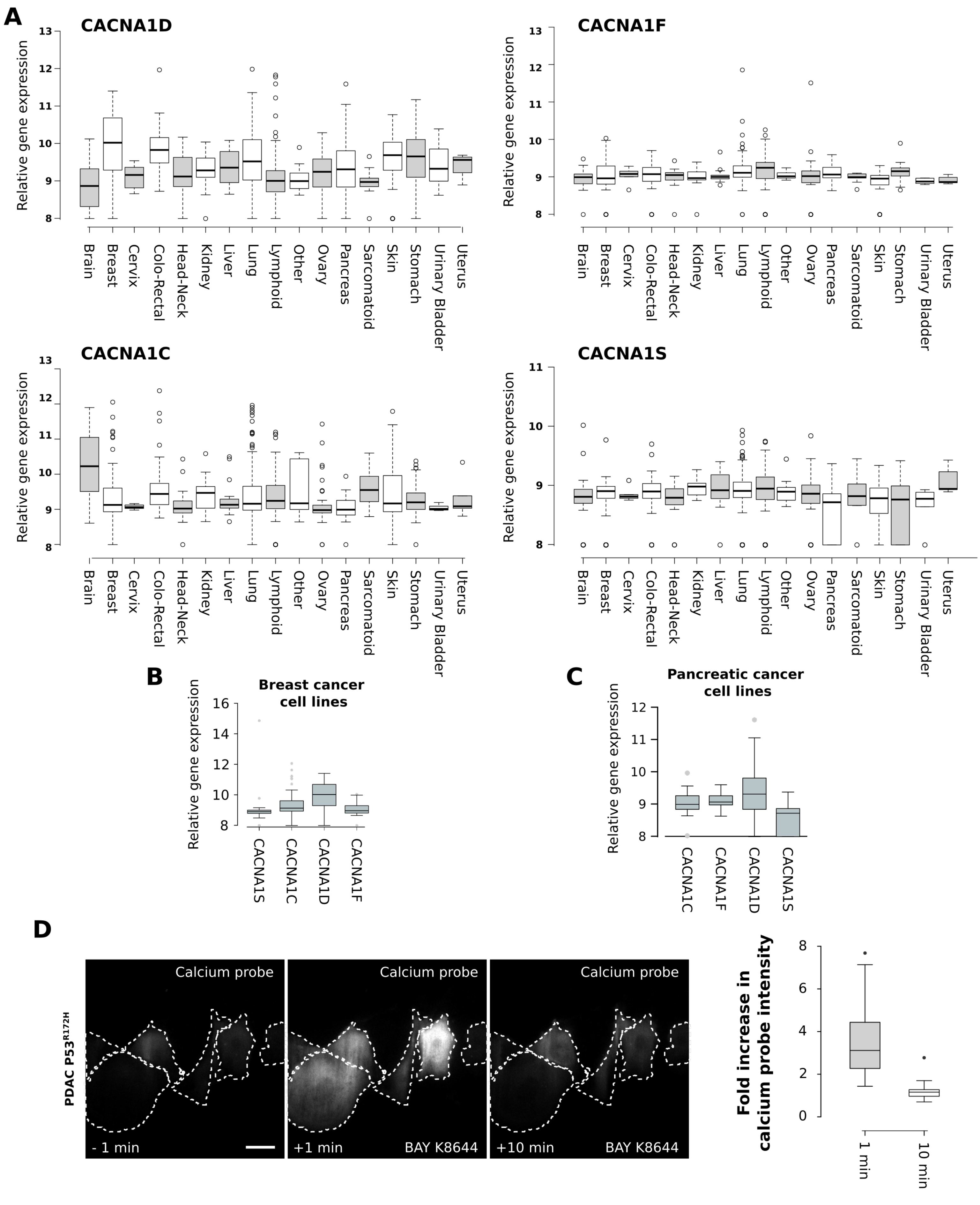

Supplementary Figure 3: L-type calcium channels are commonly expressed and functional in cancer cell lines

A: Relative expression of the four genes encoding the L-type calcium channel α1 subunits across 676 commonly used cancer cell lines organized by tissue Supergroup (number of cell lines per tissue Supergroup: Brain, 35; Breast, 70; Cervix, 6; Colo-Rectal, 55; Head-Neck, 24; Kidney, 14; Liver, 16; Lung, 150; Lymphoid, 105; Other, 10; Ovary, 48; Pancreas, 38; Sarcomatoid, 9; Skin, 54; Stomach, 30; Urinary Bladder, 6; Uterus, 5). Data presented are the gene expression read counts. The value 7.99 corresponds to non-detected. **B-C:** Relative expression of the four L-type calcium channel α1 subunits across commonly used breast (B) or pancreatic (C) cancer cell lines. **D:** P53R172H PDAC cells transiently expressing the calcium probe (pGP-CMV-GCaMP6s) and adhering to FN were treated with an L-type calcium channel activator (BAY K8644; 1 µM) while being imaged on a TIRF microscope. The relative increase in intracellular calcium concentration was measured at 1 min and 10 min post stimulation (n = 74 cells, three biological repeats; scale bar = 20 µm).

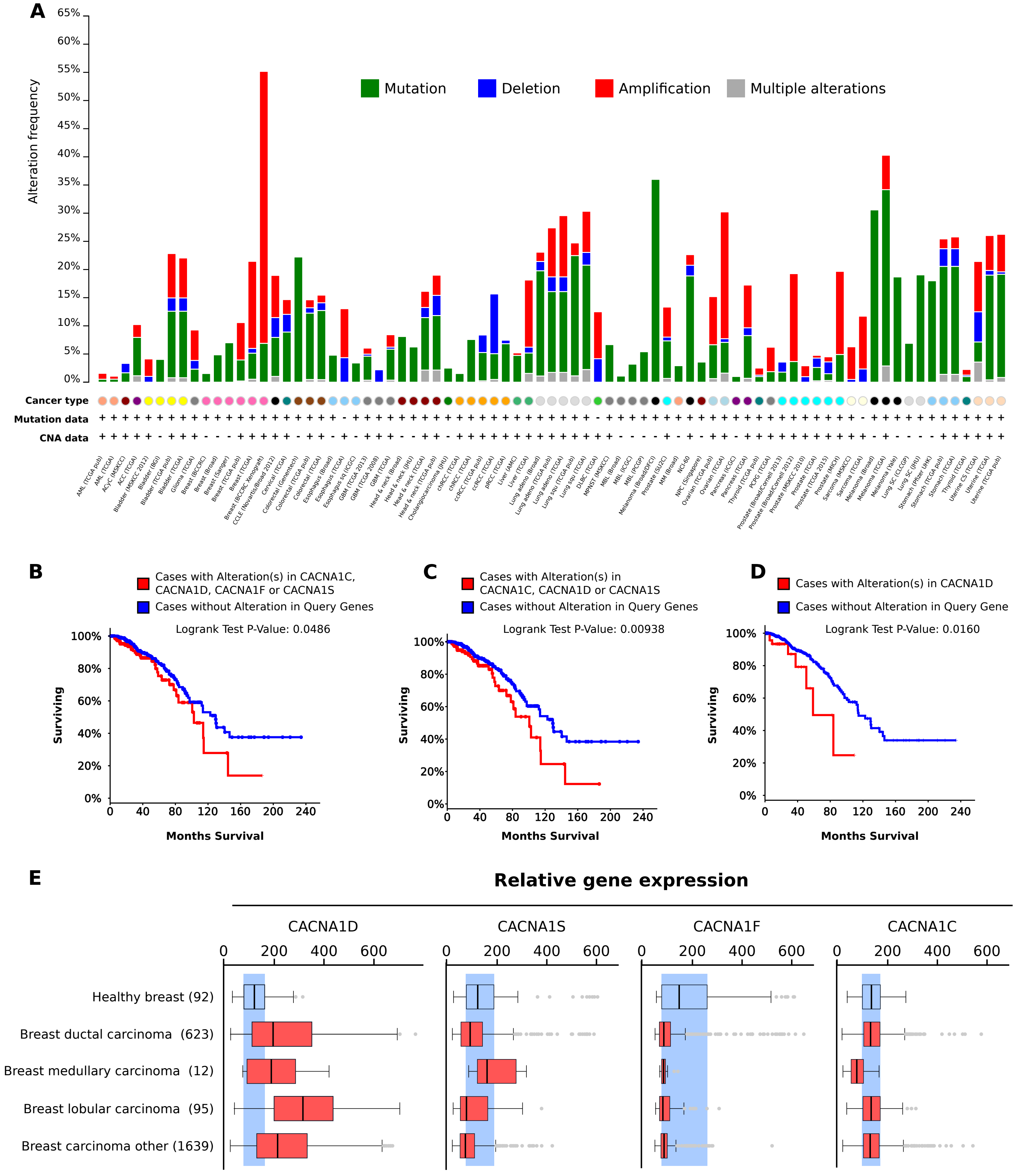

Supplementary Figure 4: L-type calcium channels are commonly expressed in cancer patient samples
A: cBioPortal analysis displaying the frequency of genetic alteration(s) (mutation, deletion, amplification) in at least one of the genes encoding the L-type calcium channel α1 subunit (CACNA1C, CACNA1D, CACNA1F, CACNA1S) in various publicly available datasets. **B:** Effect of genetic alteration(s) in at least one the genes encoding the L-type calcium channel α1 subunit (CACNA1C, CACNA1D, CACNA1F, CACNA1S) on breast cancer patient survival (Breast Invasive Carcinoma dataset (TCGA, Cell 2015)). **C:** Effect of genetic alteration(s) in CACNA1C, CACNA1D, CACNA1S on breast cancer patient survival (Breast Invasive Carcinoma dataset (TCGA, Cell 2015)). **D:** Effect of genetic alteration(s) in CACNA1D on breast cancer patient survival (Breast Invasive Carcinoma dataset (TCGA, Cell 2015)). E: Relative CACNA1D, CACNA1S, CACNA1C and CACNA1F gene expression in healthy breast or in breast tissue obtained from patients diagnosed with various types of breast cancer (IST Online database; Medisapiens Ltd).

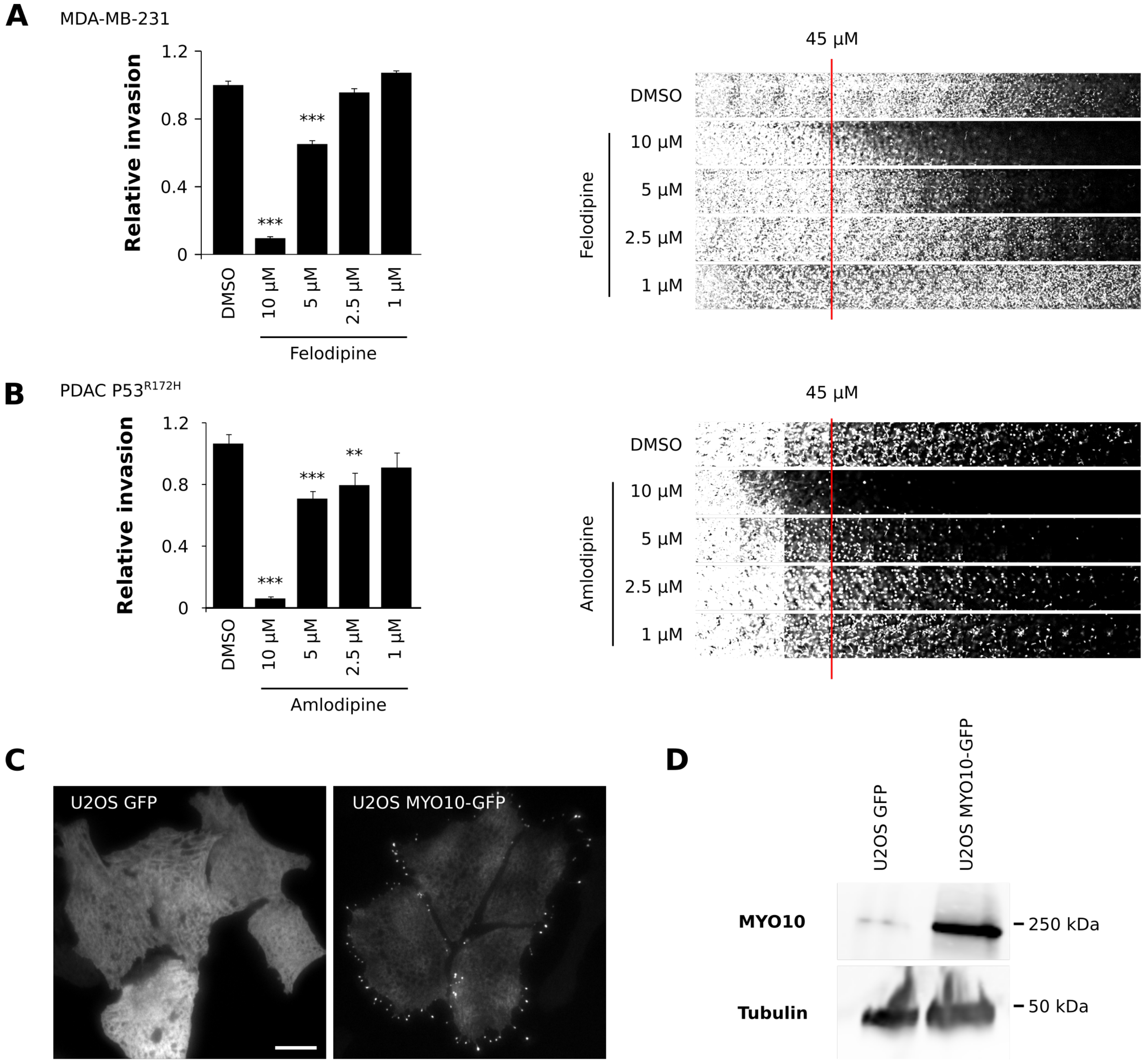

Supplementary Figure 5: CCBs inhibit cancer cell invasion and directional cell migration

A: MDA-MB-231 cells were seeded into an inverted invasion assay in the presence of decreasing concentrations of felodipine for 48 h. Relative invasion over 45 µm was quantified (n = three biological repeats, *** p value < 3.4×10^{-9}). **B:** P53R172H PDAC cells were seeded into an inverted invasion assay in the presence of decreasing concentrations of amlodipine besylate for four days. The relative invasion over 45 µm was quantified (n = three biological repeats, ** p value = 0.012, *** p value < 4.5×10^{-5}). **C:** U2OS stably expressing GFP or MYO10-GFP were plated on FN and imaged live on a TIRF microscope. **D:** U2OS stably expressing GFP or MYO10-GFP were lysed and the levels of MYO10 were analysed by western blot. The uncropped blots are available in Supplementary Fig. 12.

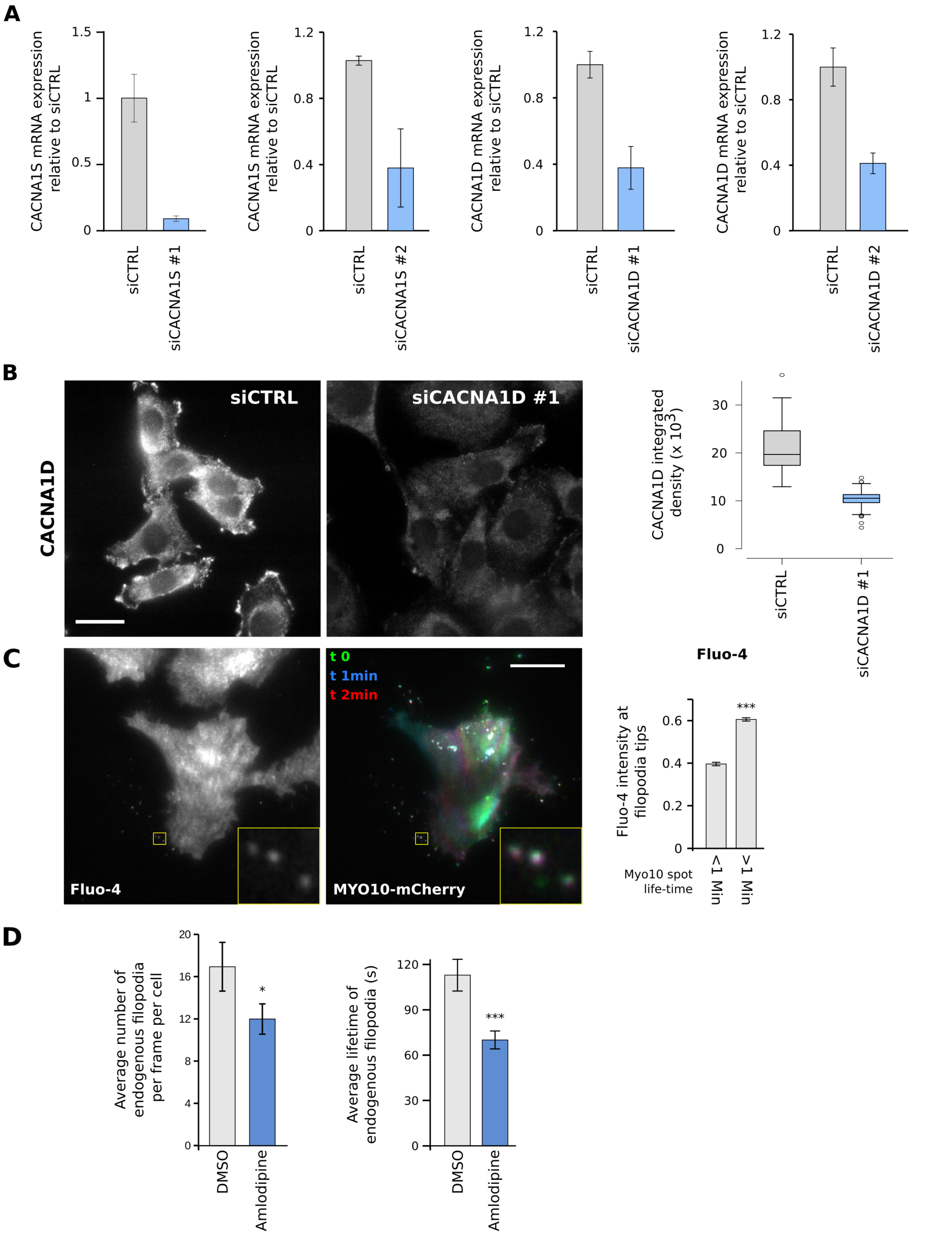

Supplementary Figure 6: Validation of siRNA-mediated knockdown of CACNA1D and CACNA1S and calcium and filopodia stability

A: Relative CACNA1D or CACNA1S expression determined by Q-RT-PCR in MDA-MB-231 cells pretreated with different siRNA oligos targeting CACNA1D or CACNA1S. **B**: MDA-MB-231 cells previously silenced for CACNA1D were plated on FN and stained for CACNA1D (scale bar = 20 µm). Average CACNA1D integrated density per cell was measured using ImageJ. **C**: MDA-MB-231 cells transiently expressing MYO10-mCherry were plated on FN, incubated with Fluo4-AM for 1 h and imaged live using a TIRF microscope. The quantification was performed as in Fig. 4A (n = 408 filopodia, three biological repeats; *** p value < 1.48×10^{-16}; scale bar = 20 µm). **D**: P53R172H PDAC cells transiently expressing lifeact-GFP were plated on FN, treated with DMSO or amlodipine besylate (10 µM), and imaged live on a TIRF microscope. Movies were segmented and filopodia identified automatically using CellGeo (See methods for details). Average number of filopodia per frame and per cell and average filopodia lifetimes are displayed (n > 19 cells, three biological repeats; * p value = 0.044, *** p value < 1.1×10^{-4}).

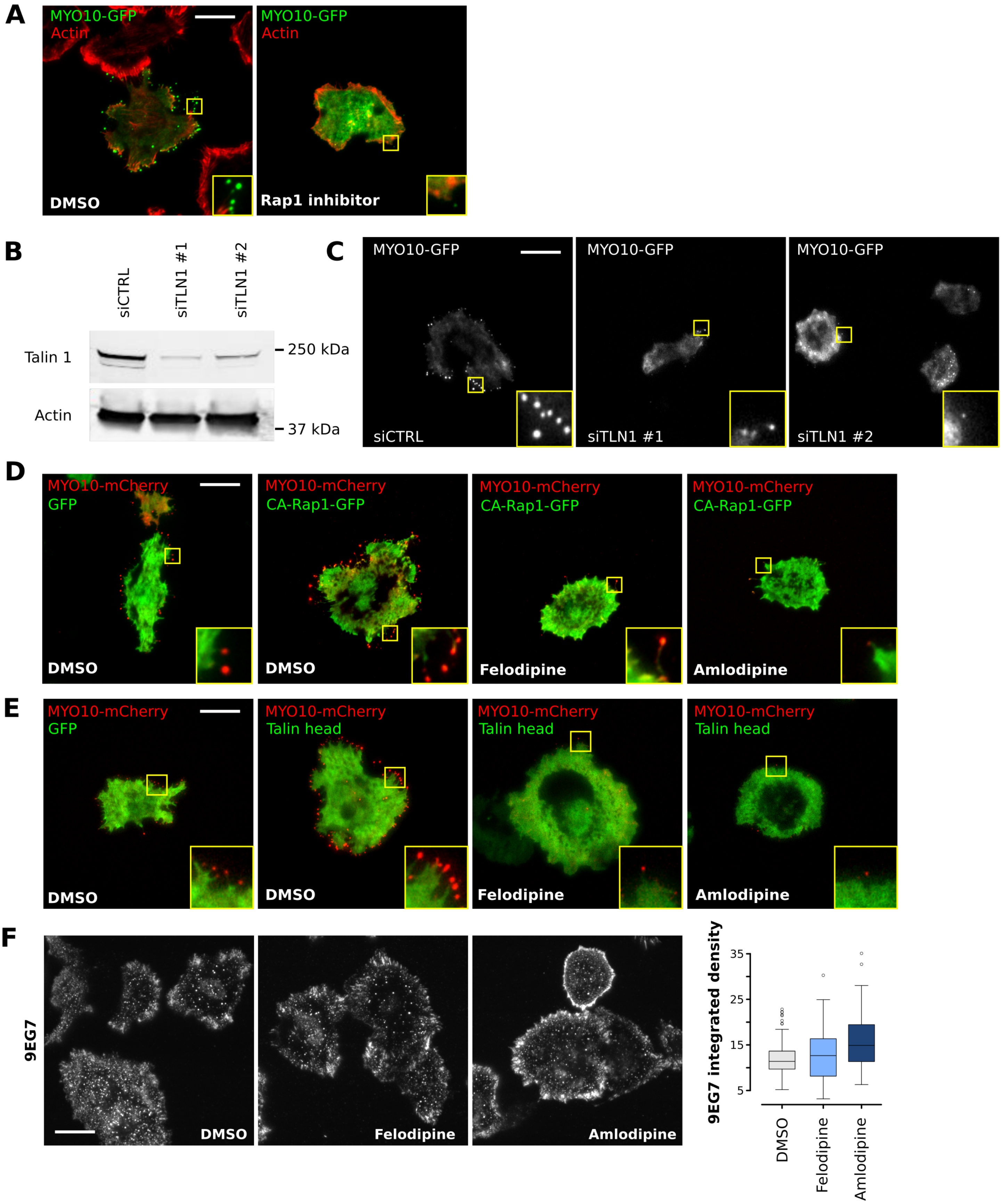

Supplementary Figure 7: Integrin inside-out signalling regulates filopodia formation.

A: MDA-MB-231 cells transiently expressing MYO10-GFP were plated on FN and treated for 1h with a Rap1 inhibitor (GGTI 298, 10 µM; scale bar= 20 µm). **B**: MDA-MB-231 cells previously silenced for talin-1, using two distinct siRNA oligos, were lysed and talin-1 levels were analysed by Western blot. The uncropped blots are available in Supplementary Fig. 12. **C**: MDA-MB-231 cells previously silenced for talin-1, using two distinct siRNA oligos, and transiently expressing MYO10-GFP were plated on FN for 2 h (scale bar = 20 µm). **D**: MDA-MB-231 cells transiently expressing MYO10-mCherry together with GFP or with CA-Rap1-GFP were plated on FN, treated with DMSO, felodipine or amlodipine besylate (10 µM; scale bar = 20 µm). **E**: MDA-MB-231 cells transiently expressing MYO10-mCherry together with GFP or with GFP tagged talin head were plated on FN and treated with DMSO, felodipine or amlodipine besylate (10 µM; scale bar = 20 µm). **F**: MDA-MB-231 cells were plated on FN, treated with DMSO, felodipine or amlodipine besylate (10 µM), stained for active β1 integrin (9EG7 clone) and imaged on a TIRF microscope (scale bar = 20 µm). β1 integrin activity was quantified by measuring the integrated density of the active integrin staining ($n > 195$ cells, three biological repeats).

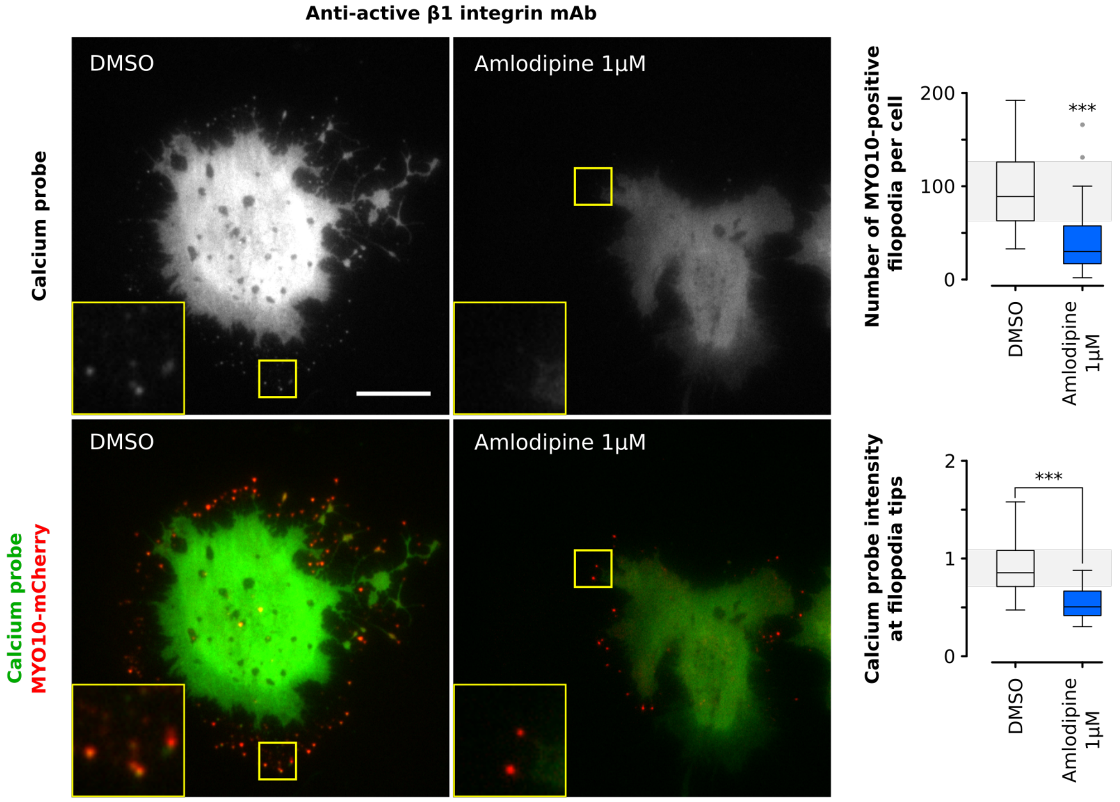

Supplementary Figure 8: CCB treatment inhibits integrin-mediated calcium entry at filopodia.
MDA-MB-231 cells transiently expressing MYO10-mCherry and the calcium probe were plated on the anti-active β1 integrin antibody (12G10) for 2 h in the presence of DMSO or 1 µM amlodipine besylate. Representative images are displayed (scale bar = 20 µm). For each conditions, the number of MYO10-positive filopodia per cell and the calcium probe intensity at filopodia tips were measured (n > 85 cells, three biological repeats; *** p value < 3.2×10^{-13}).

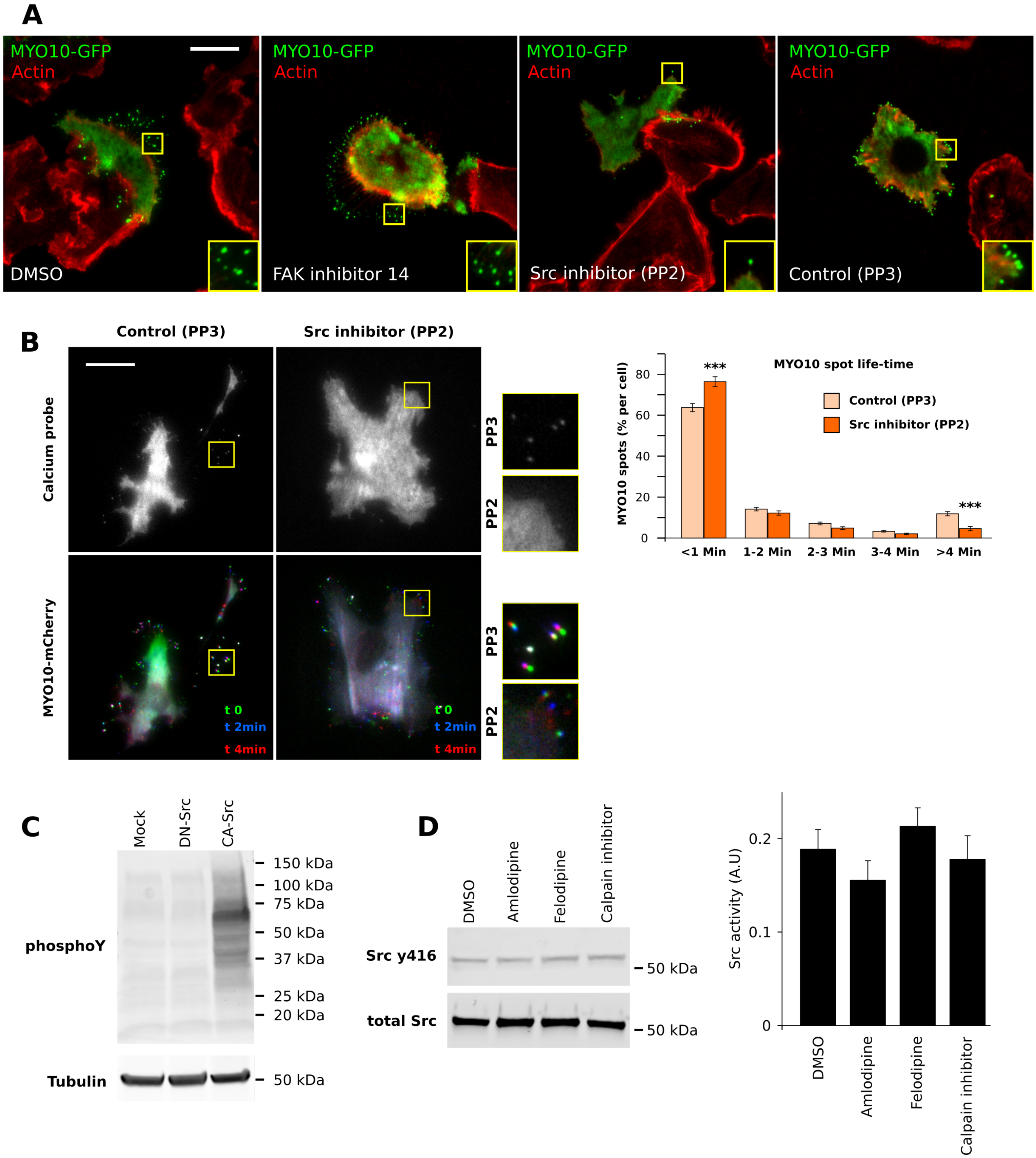

Supplementary Figure 9: Src activity regulates filopodia formation

A: MDA-MB-231 cells transiently expressing MYO10-GFP were plated on FN and treated for 1 h with DMSO, a FAK inhibitor (FAK inhibitor 14; 10 µM), a Src inhibitor (PP2; 10 µM) or a negative control associated with the Src inhibitor (PP3; 10 µM; scale bar= 20 µm). **B**: MDA-MB-231 cells transiently expressing the calcium probe (pGP-CMV-GCaMP6s) and MYO10-mCherry were plated on FN, treated with a Src inhibitor (PP2; 10 µM) or a negative control associated with the Src inhibitor (PP3; 10 µM) and imaged live using a TIRF microscope (scale bar = 20 µm). For each condition, MYO10-positive particles were automatically tracked, and their lifetime plotted as a percentage of the total number of filopodia generated per cell (see methods for details; n > 4000 particles tracked in more than 16 cells across three biological repeats; *** p value < $2x10^{-4}$). **C**: MDA-MB-231 cells transiently expressing PCDNA3 (mock), dominant negative Src (DN-SRC; Src K295R Y527F) or constitutively active Src (CA-Src; Src E378G) were lysed and the over-all phosphotyrosine levels were analysed by western blot. The uncropped blots are available in Supplementary Fig. 12. **D**: MDA-MB-231 cells were plated on FN and treated with DMSO, felodipine, amlodipine besylate or a calpain inhibitor for 1 h, lysed and the levels of total Src and of phospho Y416 Src were measured by Western blot. Src activity was quantified as a ratio of levels of phospho Y416 Src divided by total Src (n = 2). The uncropped blots are available in Supplementary Fig. 12.

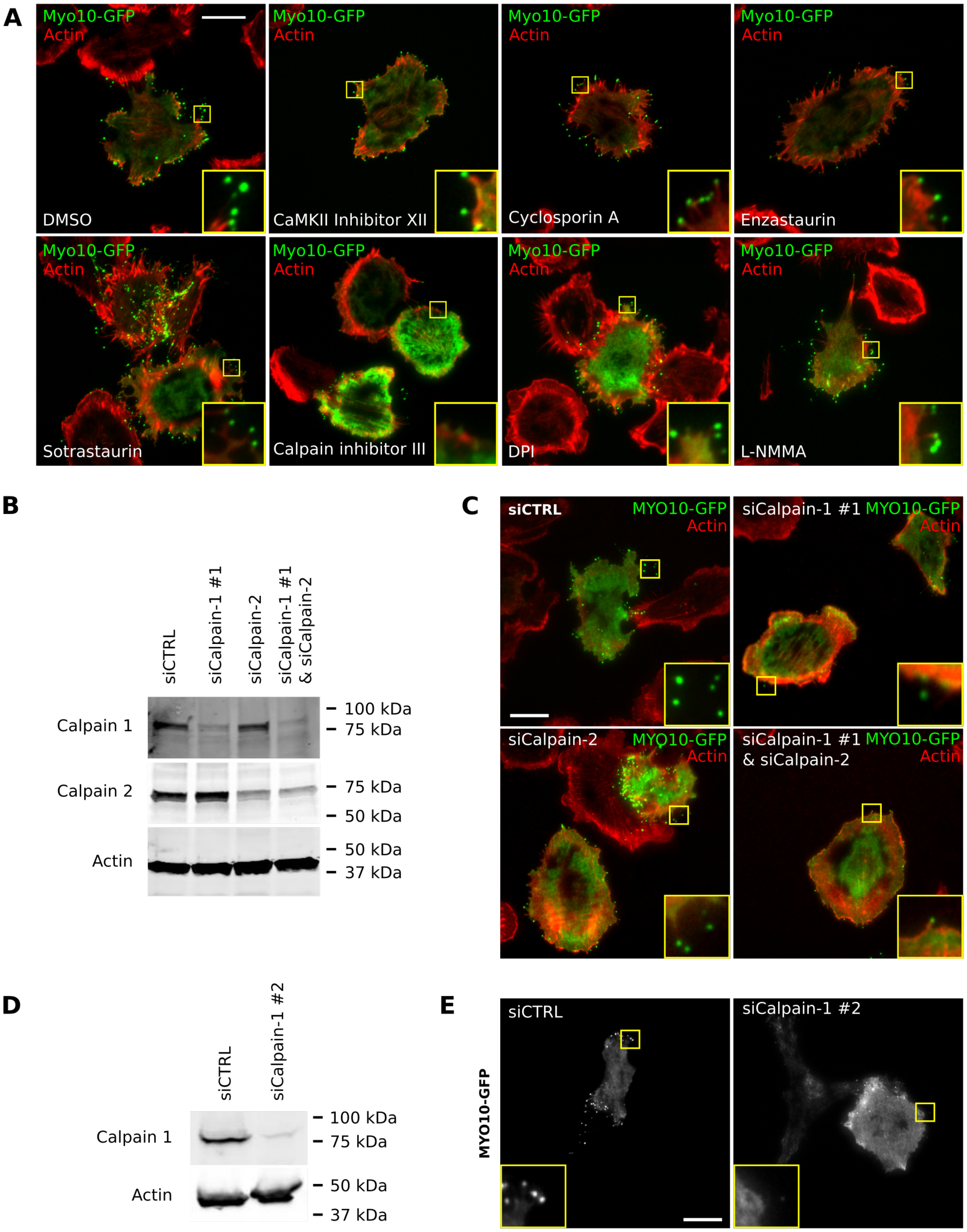

Supplementary Figure 10: Calpain-1 regulates filopodia formation

A: MDA-MB-231 cells transiently expressing MYO10-GFP were plated on FN, treated for 1 h with various compounds inhibiting calcium-regulated pathways (10 µM with the exception of Cyclosporin A, Enzastaurin and Sotrastaurin used at 1 µM), fixed and imaged on a TIRF microscope (scale bar = 20 µm). **B**: MDA-MB-231 cells previously silenced for calpain-1 or calpain-2 were lysed and calpain-1 and calpain-2 levels were analysed by Western blot. The uncropped blots are available in Supplementary Fig. 13. **C**: MDA-MB-231 cells previously silenced for calpain-1 and/or calpain-2 and transiently expressing MYO10-GFP were plated on FN for 2 h (scale bar = 20 µm). **D**: MDA-MB-231 cells previously silenced for calpain-1, using a different oligo than in (A), were lysed and calpain-1 levels were analysed by Western blot. The uncropped blots are available in Supplementary Fig. 12. **E**: MDA-MB-231 cells previously silenced for calpain-1, using a different oligo than in (A), and transiently expressing MYO10-GFP were plated on FN for 2 h (scale bar = 20 µm).

A

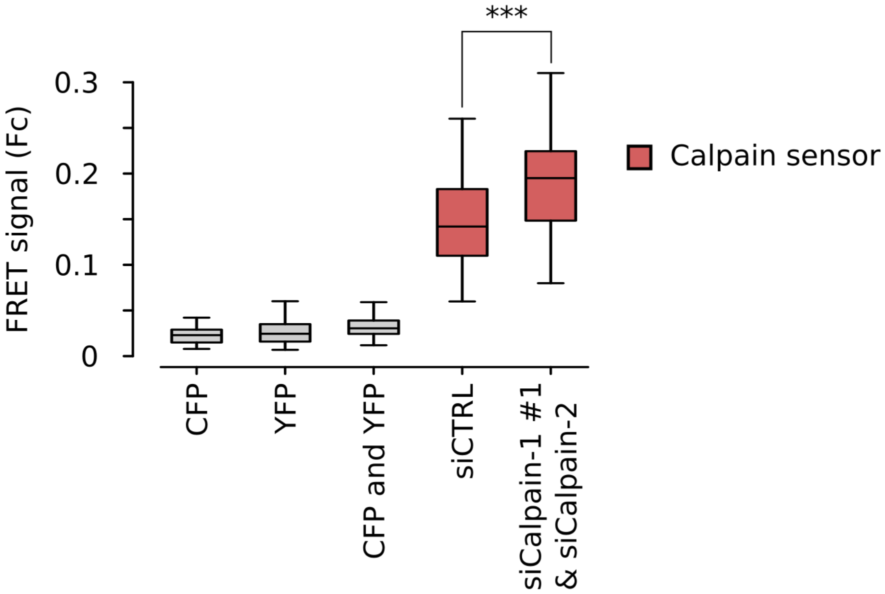

B

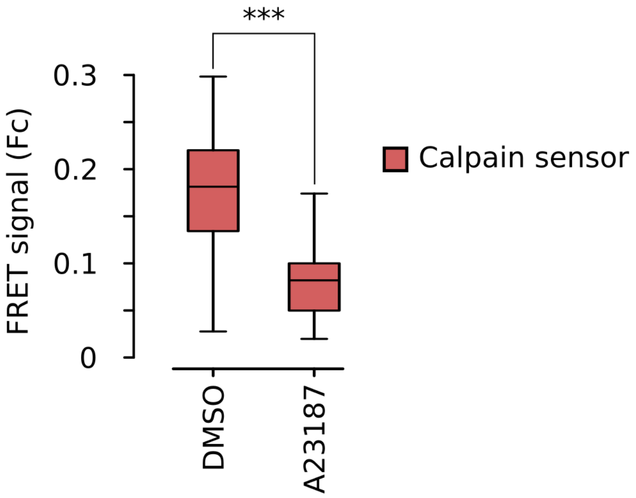

Supplementary Figure 11: Controls related to the FRET experiment presented in figure 7.
A: MDA-MB-231 cells, previously silenced for calpain 1 and calpain 2, or treated with control oligo and transiently expressing CFP (donor only), YFP (acceptor only), CFP and YFP (free), or a calpain FRET probe (pCMV-calpainsensor; CFP and YFP linked by a calpain cleavage site, low FRET = higher calpain activity) were plated on FN and imaged on a confocal microscope. The averaged FRET signals measured in the cell body are displayed ($n > 34$; *** p value < 8.9×10^{-15}). **B**: MDA-MB-231 cells transiently expressing a calpain FRET probe (pCMV-calpainsensor) and plated on FN were treated with DMSO or the calcium ionophore Calcimycin (to trigger calpain activation; A23187; 10 μM) were imaged on a confocal microscope. The averaged FRET signals measured in the cell body are displayed ($n > 38$; *** p value < 5.8×10^{-11}).

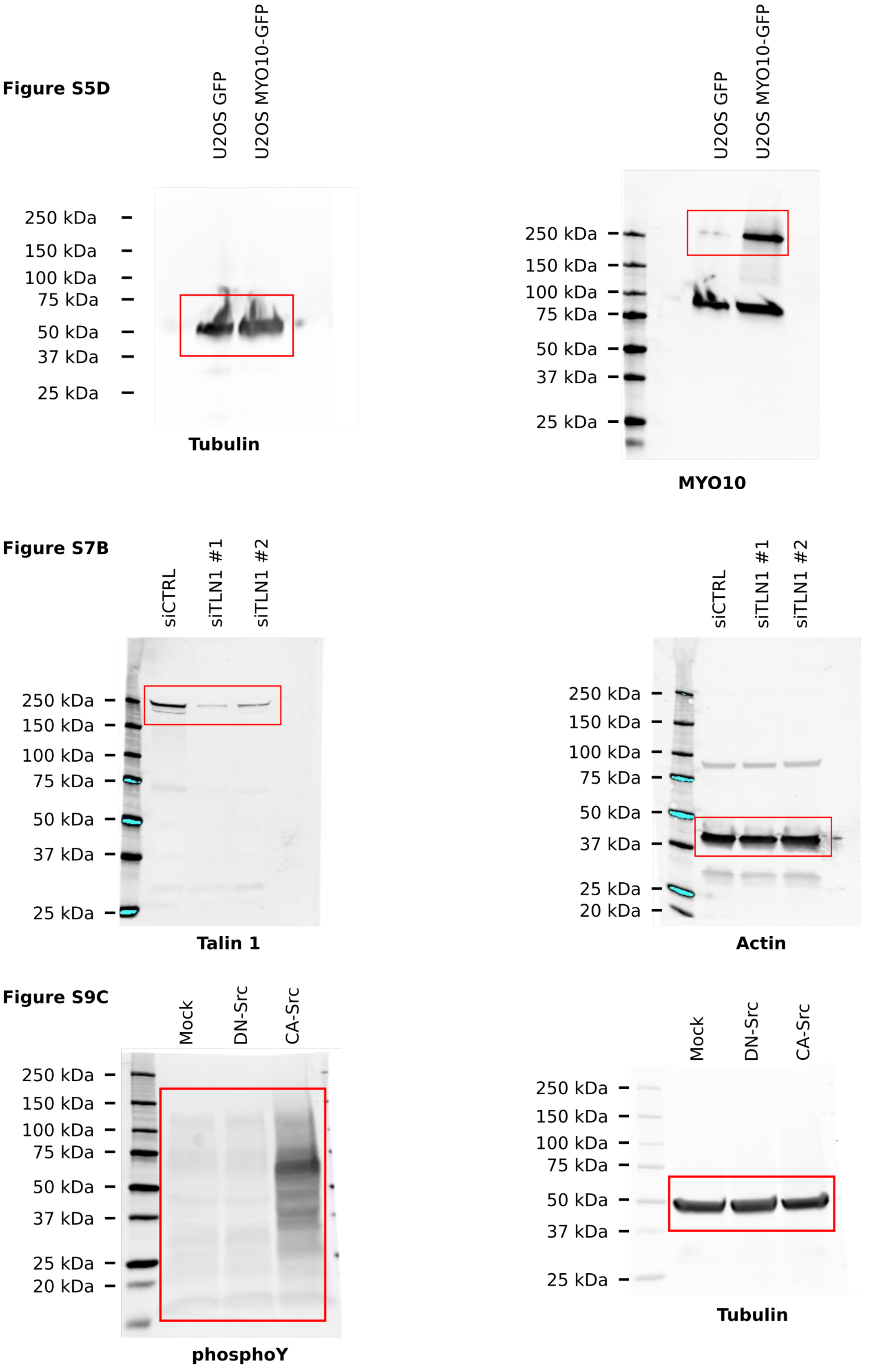

Supplementary Figure 12: Complete blots part 1
The blots displayed in Supplementary Fig. 5D, Supplementary Fig. 7B and Supplementary Fig. 9C are shown here in full. Red rectangles indicate the cropped region shown in each figure.

Figure S9D

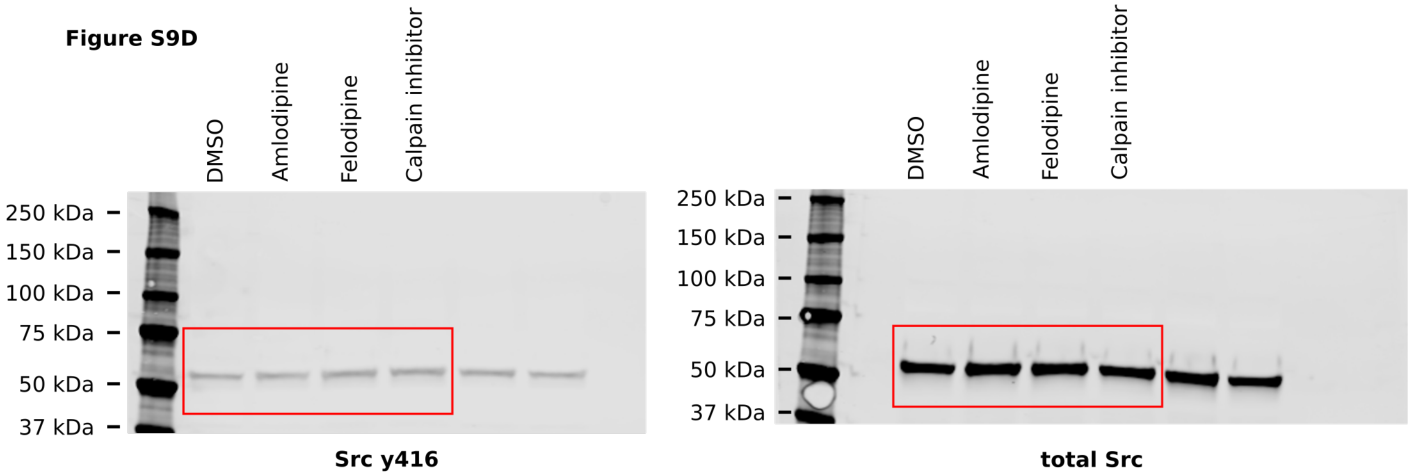

Figure S10B

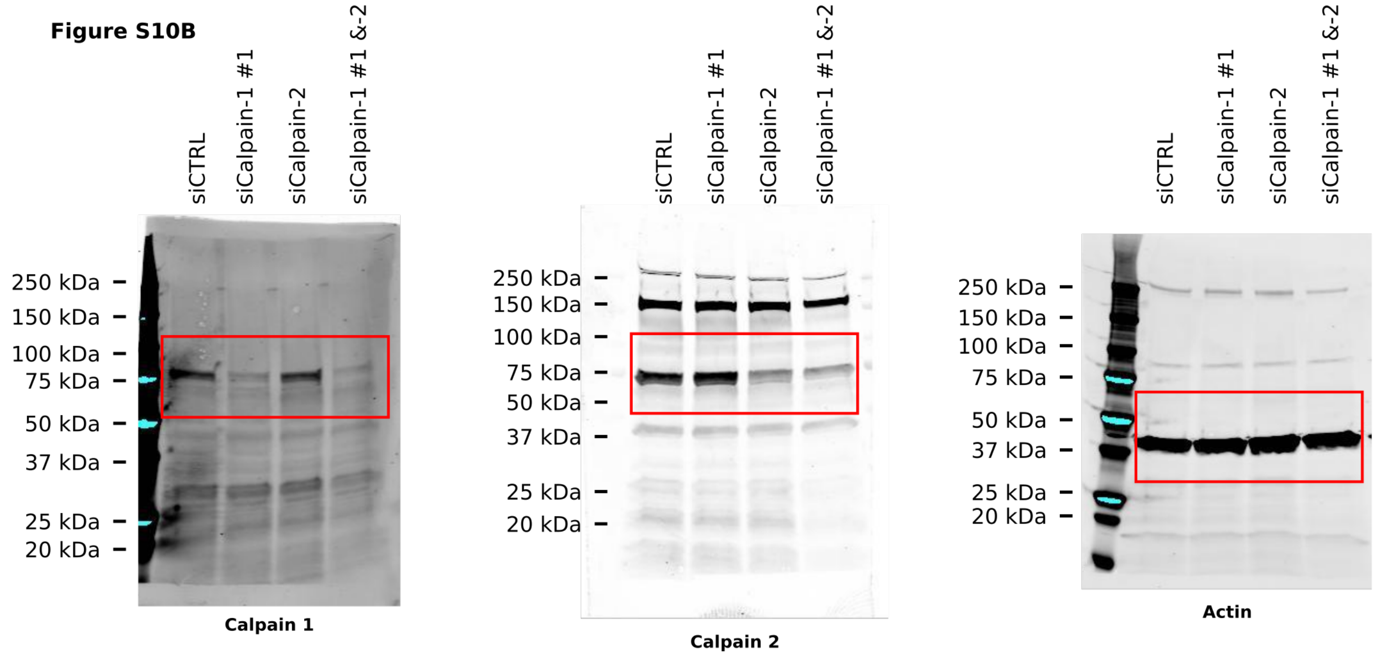

Figure S10D

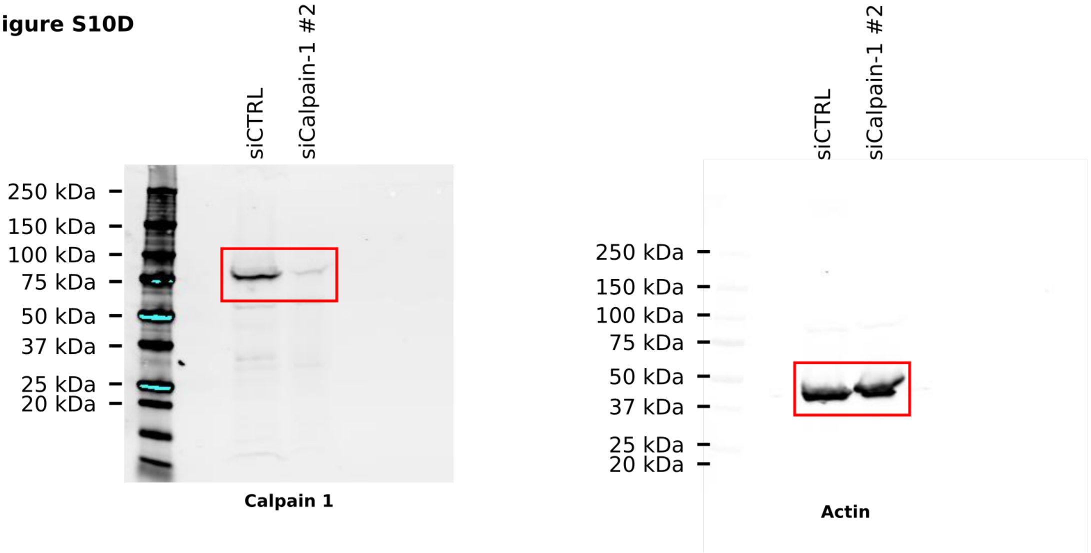

Supplementary Figure 12: Complete blots part 2
The blots displayed in Supplementary Fig. 9D, Supplementary Fig. 10B and Supplementary Fig. 10D are shown here in full. Red rectangles indicate the cropped region shown in each figure.

Supplementary Table 1

	Cases with Alteration(s) in Query Gene(s)			Cases without Alteration(s) in Query Gene(s)			
**Query Gene(s)**	**#total cases**	**#cases deceased**	**median months survival**	**#total cases**	**#cases deceased**	**median months survival**	**Logrank Test P-Value**
CACNA1C	79	10	114.72	871	101	113.73	0.992
CACNA1D	57	8	58.84	893	103	114.72	0.016
CACNA1F	58	6	NA	892	105	114.06	0.467
CACNA1S	121	16	100.62	829	95	114.72	0.148
CACNA1C, CACNA1D	129	18	114.06	821	93	122.8	0.115
CACNA1C, CACNA1F	126	15	114.72	824	96	113.73	0.817
CACNA1C, CACNA1S	189	26	102.69	761	85	129.47	0.0651
CACNA1D, CACNA1F	108	12	102.69	842	99	114.72	0.673
CACNA1D, CACNA1S	169	22	100.62	781	89	122.8	0.0247
CACNA1F, CACNA1S	169	20	102.69	781	91	114.72	0.436
CACNA1C, CACNA1D, CACNA1F	172	21	114.06	778	90	122.8	0.506
CACNA1C, CACNA1D, CACNA1S	231	32	100.62	719	79	129.47	0.00938
CACNA1D, CACNA1F, CACNA1S	212	25	100.62	738	86	122.8	0.172
CACNA1C, CACNA1D, CACNA1F, CACNA1S	267	34	102.69	683	77	129.47	0.0486

Supplementary Table 2: Compounds used in this study

Compound name	Alternative name	Provider	Target	Concentration used
Cilnidipine	/	Selleckchem S1293	L-type calcium channel blocker	10 µM
Manidipine dichloride	/	Selleckchem S2482	L-type calcium channel blocker	10 µM
Felodipine	/	Selleckchem S1885, F9677 Sigma	L-type calcium channel blocker	as indicated
Amlodipine besylate	/	Selleckchem S1813, A5605 Sigma	L-type calcium channel blocker	as indicated
Zonisamide	/	Selleckchem S1445	sodium channel and T-type calcium channel blocker	10 µM
Bumetanide	/	Selleckchem S1287	sodium channel blocker	10 µM
Carbamazepine	/	Selleckchem S1693	sodium channel blocker	10 µM
LY294002	/	Selleckchem S1105	PI3K inhibitor	10 µM
(S) - (-) - Bay K8644	/	Sigma B133	L-type calcium channel activator	1 µM
Rap1 inhibitor	GGTI 298 trifluoroacetate salt hydrate	G5169 Sigma	geranylgeranyltransferase I (GGTase I) inhibitor	10 µM
FAK inhibitor 14	/	R&D Systems 3414	FAK inhibitor	10 µM
PP2	/	Selleckchem S7008	Src family kinase inhibitor	10 µM
PP3	/	Abcam ab120617	Negative control for PP2	10 µM
CaMKII Inhibitor XII	/	Merck Millipore 208923	CaMKII inhibitor	10 µM
Cyclosporin A	/	30024 Sigma	calcineurin inhibitor	1 µM

Enzastaurin	/	Selleckchem S1055	PKC inhibitor	1 µM
Sotrastaurin	/	Selleckchem S2791	PKC inhibitor	1 µM
Calpain inhibitor III	MDL 28170	M6690 Sigma	Calpain inhibitor	10 µM
DPI	Diphenyleneiodonium chloride	D2926 Sigma	inhibitor of nitric oxide synthetase	10 µM
L-NMMA	NG-Methyl-The-arginine acetate salt	M7033 Sigma	inhibitor of nitric oxide synthetase	10 µM
Calcimycin	A23187	C7522 Sigma	Calcium Ionophore	10 µM
